# Nomogram to predict the prognosis of parotid gland mucoepidermoid carcinoma: a population-based study of 1306 cases

**DOI:** 10.7717/peerj.7237

**Published:** 2019-07-01

**Authors:** Jian Sun, Yang Sun, Fei Yang, Qianrong Zhou, Wenjuan Liu, Yong Cheng, Xingwen Wu, Tinglan Chen, Ruixue Li, Borui Huang, Wael Att, Youcheng Yu, Wei Bi

**Affiliations:** 1 Department of Stomatology, Zhongshan Hospital, Fudan University, Shanghai, China; 2 Department of Stomatology, Xuhui Central Hospital, Shanghai, China; 3 Department of Prosthodontics, School of Dentistry, Albert Ludwigs University, Freiburg, Germany; 4 Department of Prosthodontics, Dental Medicine, Tufts University School, Boston, United States of America

**Keywords:** Mucoepidermoid carcinoma, Parotid gland, Prognosis, Nomogram, SEER database

## Abstract

**Background:**

Mucoepidermoid carcinoma (MEC) is a common cancer in the oral salivary gland malignancy, which mainly occurs in the parotid gland. The aim of this study is to identify independent prognostic factors and establish a nomogram model for parotid gland mucoepidermoid carcinoma (P-MEC) patients using the National Cancer Institute’s Surveillance, Epidemiology, and End Results (SEER) database.

**Method:**

Patients with P-MEC were selected from between 2004 and 2015. The overall survival (OS) and cancer-specific survival (CSS) rates were estimated using the Kaplan-Meier method with the log-rank test. Univariate and multivariate Cox proportional hazards regression analyses were performed to identify the independent prognostic factors.

**Results:**

A total of 1,306 patients with P-MEC were enrolled. Age, grade, T stage, N stage, M stage, chemotherapy, and surgery type were independent prognostic factors for OS and CSS. A nomogram for OS was formulated based on these independent prognostic factors and validated using an internal bootstrap resampling approach, which showed that the nomogram exhibited a sufficient level of discrimination according to the C-index (0.877, 95% CI [0.855–0.898]).

**Conclusion:**

Several prognostic factors for P-MEC were identified. The nomogram developed in this study accurately predicted the 5- and 10-year OS rates of American patients with P-MEC based on individual characteristics. Risk stratification using the survival nomogram can optimize individual therapies and follow-up.

## Introduction

Mucoepidermoid carcinoma (MEC) is the most common salivary gland malignancy. About 50% MEC occurs in the major salivary glands, with 70% in the parotid gland (Rajasekaran et al., 2018; Chen et al., 2014). Surgery is still the mainstay of treatment for malignant salivary gland tumors. The selection of surgical procedure depends on the location and grade of the tumor. Most parotid tumors are confined to the superficial lobe and require a superficial parotidectomy, while only 10% to 20% of tumors involve the deep lobe, which requires a total gland resection (Lewis, Tong & Maghami, 2016; Patel et al., 2015). Postoperative radiotherapy has shown a survival benefit in patients with major salivary gland carcinoma. Indications of postoperative radiotherapy include positive or close margin (<0.5 cm), high grade, skin or bone infiltration, perineural invasion and lymph node metastases. Chemotherapy is mainly used in the palliative setting. However, even after surgical resection and postoperative radiotherapy, 40% of cases relapse (Kaur et al., 2014).

The prognostic factors of parotid gland mucoepidermoid carcinoma (P-MEC) included sex, age, histological grade and surgical margins (Rajasekaran et al., 2018; Kandaz et al., 2016). Usually, low grade P-MEC patients exhibit a better survival than high grade P-MEC patients. However, survival is also good even in high grade P-MEC patients diagnosed with an early stage malignancy (Liu et al., 2014). Ghosh-Laskar et al. (2011) reported a better overall survival in P-MEC patients who were 54 years old or younger, female, non-white , with no comorbidities according to the Charlson/Deyo score, suffering from low grade P-MEC, with an early stage tumor and who had a negative surgical margin. To date, large-scale studies on the prognostic factors for P-MEC are limited and the reported associations have not been confirmed (Ghosh-Laskar et al., 2011).

Nomography has been widely used to predict the survival of cancer patients (Kattan et al., 1998; Wang et al., 2018; Yin et al., 2018). However, a nomogram for P-MEC has not been developed. Therefore, we constructed a nomogram to visually predict the 5- and 10-year overall survival rate of P-MEC patients using data from the Surveillance, Epidemiology, and End Results (SEER) database. We expect that our research will improve the understanding of P-MEC and optimize individual therapies and follow-up.

## Patients and Methods

### Ethics statement

Approval for use of all data was obtained by submitting a request to the SEER program. No approval by the institutional review board was sought since SEER is a public database.

### Patients

Data were extracted using SEER*Stat software (version 8.3.5) from the SEER Program (http://www.seer.cancer.gov) SEER*Stat Database: Incidence - SEER 18 Regs Custom Data (with additional treatment fields), Nov 2017 Sub (1973–2015, varying) - Linked To County Attributes - Total U.S., 1969–2016 Counties, National Cancer Institute, DCCPS, Surveillance Research Program, released April 2018, based on the November 2017 submission.

**Figure 1:**
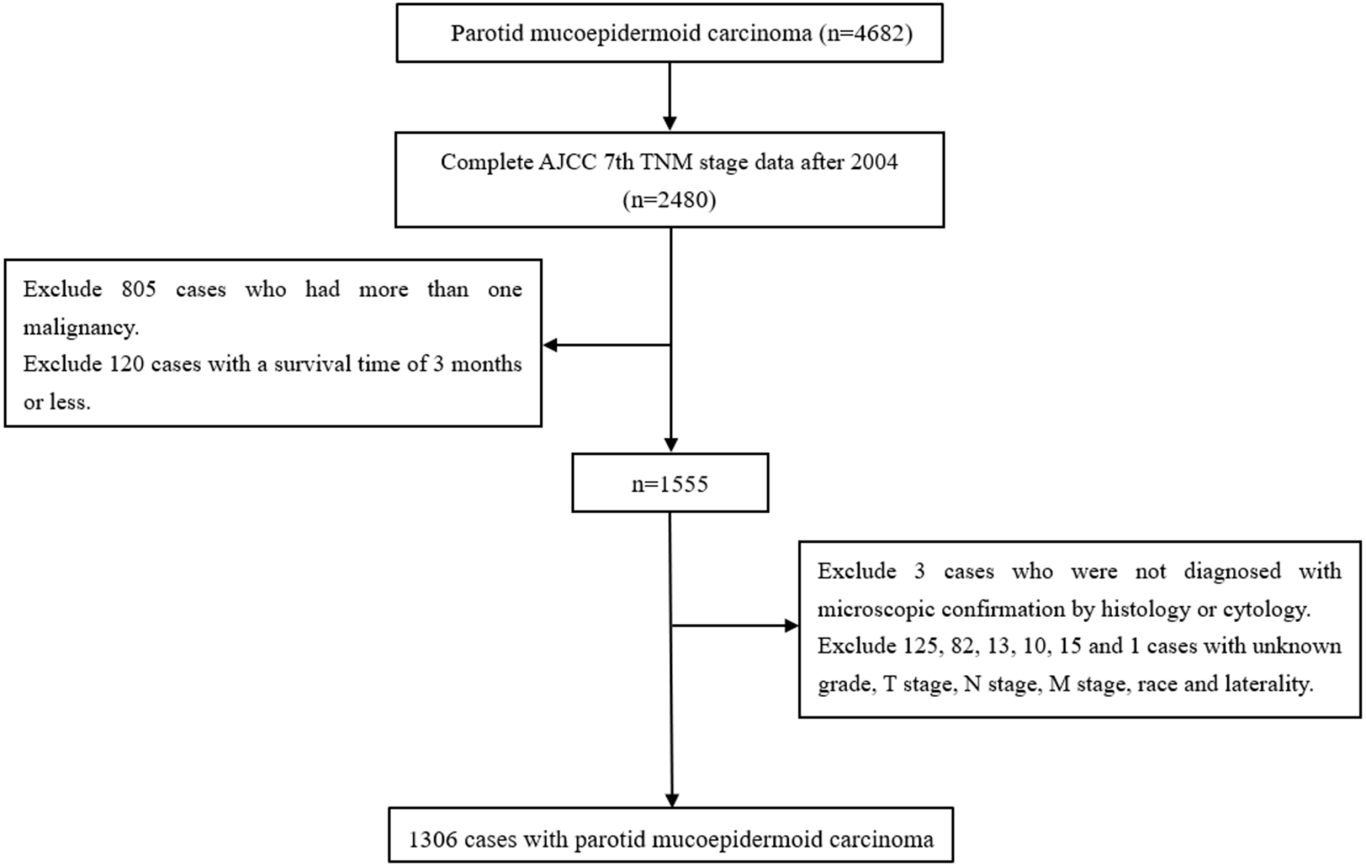
The flow diagram of the selection process for the study cohort.

Cases in which the primary site of the parotid gland were selected using the variable “primary site” (parotid gland = 079). Cases in which P-MEC were identified by histology (International Classification of Diseases code: 8430) (Chen et al., 2014). Cases were excluded if: the AJCC 7th TNM stage data were not complete; they had more than one malignancy, a survival time of three months or less, were not diagnosed with microscopic confirmation by histology or cytology and the grade, T stage, N stage, M stage, race, and laterality were unknown (Kim & Thomas, 2015; Lim, Kim & Wu, 2015). Detailed information regarding age, sex, laterality, race, marital status, grade, radiotherapy, chemotherapy, surgery, and the AJCC 7th TNM stage were extracted (Fig. 1).

### Statistical analysis

The survival time was calculated from diagnosis to death or to the end of the study period. Overall survival (OS) and cancer-specific survival (CSS) rates were estimated using the Kaplan–Meier method. Survival curves were compared using the log-rank test for significance. Univariate and multivariate hazard analysis was conducted using the cox proportional hazards model to identify independent prognostic factors. The power analysis was completed with PASS (version 11). All other statistical analyses were performed using SPSS (version 24; IBM, Armonk, NY, USA). A probability value (*p* value) of <0.05 was considered statistically significant for all tests. All tests were two-sided.

### Nomogram Construction and Validation

We built a nomogram based the results of multivariate hazard analysis via the rms package of R, version 3.4.4 (R Core Team, 2018). The maximum score for each variable was set at 10. Accuracy of the nomogram was assessed based on the Harrel concordance index (C-index) and calibration curves were calculated by regression analysis to compare the nomogram-predicted and Kaplan–Meier estimated survival probability. Bootstraps of 1,000 re-samples were set.

## Results

### Patient clinicopathological data

A total of 1,306 cases of P-MEC diagnosed between 2004 and 2015 were enrolled. Demographic information is provided in Table 1. The median age was 53 years with a standard deviation of 19.6 years. Among the 1,306 cases, 660 (50.5%) patients were female, 680 (52.1%) patients had tumors originating from the right parotid gland, and 1,286 (98.5%) patients received surgery. Additionally, 609 (46.6%) and 81 (6.2%) patients received radiotherapy and chemotherapy, respectively. As for the AJCC 7th staging information, there were 573 (43.8%) cases with stage I, 296 (22.7%) cases with stage II, 200 (15.3%) cases with stage III,194 (14.9%) cases with stage IVA, 25 (1.9%) cases with stage IVB, and 18 (1.4%) cases with stage IVC P-MEC. Concerning grade, 362 (27.7%) cases were well differentiated (grade I), 608 (46.5%) cases were moderately differentiated (grade II), 168 (12.9%) cases were poorly differentiated (grade III), and 168 (12.9) cases were undifferentiated (grade IV).

### Survival analysis

The median follow-up period was 54 months (4–143 months). The 5-year OS and CSS were 83.1% and 88.7%, respectively. The 10-year OS and CSS were 73.6% and 86.4%, respectively.

**Figure 2:**
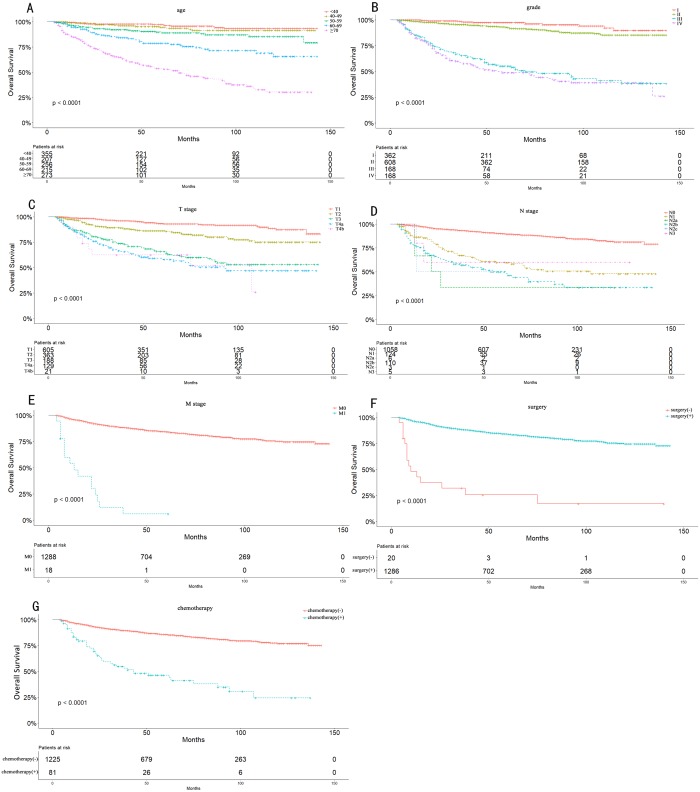
Overall Kaplan–Meier survival curves according to age (A), grade (B), T stage (C), N stage (D), M stage (E), surgery type (F) and chemotherapy (G).

Univariate analysis showed that age (*p* < 0.001), sex (*p* < 0.001), race (*p* = 0.006), grade (*p* < 0.001), TNM stage (*p* < 0.001), T stage (*p* < 0.001), N stage (*p* < 0.001), M stage (*p* < 0.001), radiotherapy (*p* < 0.001), chemotherapy (*p* < 0.001), and surgery (*p* < 0.001) were significant prognostic factors of OS (Table 2). Furthermore, age (*p* < 0.001), sex (*p* < 0.001), race (*p* = 0.010), grade (*p* < 0.001), TNM stage (*p* < 0.001), T stage (*p* < 0.001), N stage (*p* < 0.001), M stage (*p* < 0.001), radiotherapy (*p* < 0.001), chemotherapy (*p* < 0.001), and surgery (*p* < 0.001) were significant prognostic factors for CSS (Table 3). Related clinic pathological factors with p-values (*p* < 0.05) in the univariate analyses were adjusted for multivariate analysis. After multivariate analysis, only age (*p* < 0.001), grade (*p* < 0.001), T stage (*p* < 0.001), N stage (*p* < 0.001), M stage (*p* = 0.002), chemotherapy (*p* = 0.005), and surgery (*p* = 0.011) remained independent prognostic factors for OS (Table 2). Similarly, age (*p* < 0.001), grade (*p* < 0.001), T stage (*p* = 0.002), N stage (*p* < 0.001), M stage (*p* = 0.002), chemotherapy (*p* = 0.006) and surgery (*p* = 0.043) remained independent prognostic factors for CSS (Table 3). As shown in Fig. 2, patients who were younger, with a lower grade, early stage, no chemotherapy, and surgery had a significantly better OS.

**Table 1:** Characteristics of patients with parotid mucoepidermoid carcinoma.

Characteristics	Number	Percent (%)
Age		
<40 years	355	27.182
40–49 years	207	15.850
50–59 years	256	19.602
60–69 years	215	16.462
≥70 years	273	20.904
Sex		
Male	646	49.464
Female	660	50.536
Laterality		
Left	626	47.933
Right	680	52.067
Race		
White	985	75.421
Black	174	13.323
Other	147	11.256
Marital status		
Married	705	53.982
Not married	553	42.343
Unknown	48	3.675
Grade		
I	362	27.718
II	608	46.554
III	168	12.864
IV	168	12.864
TNM stage		
I	573	43.874
II	296	22.664
III	200	15.314
IVA	194	14.854
IVB	25	1.914
IVC	18	1.380
T stage		
T1	605	46.325
T2	363	27.795
T3	188	14.395
T4a	129	9.877
T4b	21	1.608
N stage		
N0	1058	81.011
N1	124	9.494
N2a	6	0.459
N2b	110	8.423
N2c	3	0.230
N3	5	0.383
M stage		
M0	1288	98.622
M1	18	1.378
Radiotherapy		
No	697	53.369
Yes	609	46.631
Chemotherapy		
No	1225	93.798
Yes	81	6.202
Surgery		
No	20	1.531
Yes	1286	98.469
Cause of death		
Alive	1081	82.772
Cancer death	137	10.490
Non-cancer death	88	6.738

**Table 2:** Parotid mucoepidermoid carcinoma: univariate and multivariate analysis of overall survival.

	Univariate		*P*	Power value	Multivariate		*P*	Power value
	HR	95% CI			HR	95% CI		
Age			<0.001^*^				<0.001^*^	
<40 years	Reference				Reference			
40–49 years	1.433	0.663–3.098	0.361	1.000	1.055	0.478–2.329	0.895	1.000
50–59 years	2.773	1.454–5.288	0.002	1.000	2.068	1.064–4.019	0.032^*^	1.000
60–69 years	6.068	3.319–11.094	<0.001^*^	1.000	3.591	1.904–6.771	<0.001^*^	1.000
≥70 years	16.582	9.542–28.818	<0.001^*^	1.000	9.262	5.125–16.739	<0.001^*^	1.000
Sex			<0.001^*^				0.384	
Male	Reference				Reference			
Female	0.493	0.375–0.649	<0.001^*^	1.000	1.139	0.850–1.525	0.384	1.000
Laterality			0.333					
Left	Reference							
Right	0.879	0.677–1.142	0.333	1.000				
Race			0.006^*^				0.587	
White	Reference				Reference			
Black	0.575	0.359–0.921	0.021^*^	1.000	0.785	0.457–1.347	0.379	1.000
Other	0.577	0.347–0.962	0.035^*^	1.000	1.126	0.667–1.899	0.657	1.000
Marital status			0.252					
Married	Reference							
Not married	1.176	0.899–1.537	0.237	1.000				
Unknown	1.578	0.827–3.013	0.166	1.000				
Grade			<0.001^*^				<0.001^*^	
I	Reference				Reference			
II	2.233	1.216–4.102	0.010^*^	1.000	1.824	0.981–3.392	0.058	1.000
III	15.638	8.691–28.138	<0.001^*^	1.000	4.112	2.107–8.026	<0.001^*^	1.000
IV	18.027	10.038–32.375	<0.001^*^	1.000	5.236	2.687–10.204	<0.001^*^	1.000
TNM stage			<0.001^*^					
I	Reference							
II	2.032	1.250–3.305	0.004^*^	1.000				
III	5.386	3.493–8.305	<0.001^*^	1.000				
IVA	8.902	5.892–13.450	<0.001^*^	1.000				
IVB	9.067	4.456–18.452	<0.001^*^	1.000				
IVC	54.136	29.337–99.900	<0.001^*^	1.000				
T stage			<0.001^*^				<0.001^*^	
T1	Reference				Reference			
T2	2.509	1.670–3.771	<0.001^*^	1.000	1.605	1.043–2.470	0.031^*^	1.000
T3	6.401	4.307–9.513	<0.001^*^	1.000	2.690	1.729–4.184	<0.001^*^	1.000
T4a	7.813	5.174–11.797	<0.001^*^	1.000	1.844	1.145–2.970	0.012^*^	1.000
T4b	8.761	4.241–18.097	<0.001^*^	1.000	2.645	1.178–5.939	0.018^*^	1.000
N stage			<0.001^*^				<0.001^*^	
N0	Reference				Reference			
N1	4.423	3.170–6.171	<0.001^*^	1.000	1.849	1.277–2.677	0.001^*^	1.000
N2a	7.823	2.884–21.223	<0.001^*^	1.000	1.164	0.403–3.356	0.779	0.791
N2b	6.893	4.985–9.532	<0.001^*^	1.000	2.321	1.598–3.370	<0.001^*^	1.000
N2c	7.430	1.035–53.312	0.046^*^	1.000	2.838	0.381–21.121	0.308	1.000
N3	4.346	1.073–17.597	0.039^*^	1.000	1.211	0.282-5.204	0.797	0.993
M stage			<0.001^*^				0.002^*^	
M0	Reference				Reference			
M1	17.167	10.189–28.924	<0.001^*^	1.000	3.104	1.526–6.314	0.002^*^	1.000
Radiotherapy			<0.001^*^				0.722	
No	Reference				Reference			
Yes	2.541	1.922–3.360	<0.001^*^	1.000	0.940	0.666–1.325	0.722	0.958
Chemotherapy			<0.001^*^				0.005^*^	
No	Reference				Reference			
Yes	5.279	3.789–7.354	<0.001^*^	1.000	1.739	1.178–2.567	0.005^*^	1.000
Surgery			<0.001^*^				0.011^*^	
No	Reference				Reference			
Yes	0.102	0.060–0.172	<0.001^*^	1.000	0.404	0.202–0.810	0.011^*^	1.000

**Notes.**

HR hazard ratio CI confidence interval

* indicates *p* < 0.05.

**Table 3:** Parotid mucoepidermoid carcinoma: univariate and multivariate analysis of cancer specific survival.

	Univariate		*P*	Power value	Multivariate		*P*	Power value
	HR	95% CI			HR	95% CI		
Age			<0.001^*^				<0.001^*^	
<40 years	Reference				Reference			
40–49 years	1.675	0.665–4.219	0.274	1.000	0.900	0.337–2.402	0.833	1.000
50–59 years	3.151	1.435–6.921	0.004	1.000	1.698	0.740–3.895	0.212	1.000
60–69 years	6.530	3.108–13.719	<0.001^*^	1.000	2.555	1.148–5.691	0.022^*^	1.000
≥70 years	12.536	6.249–25.147	<0.001^*^	1.000	4.376	2.041–9.384	<0.001^*^	1.000
Sex			<0.001^*^				0.086	
Male	Reference				Reference			
Female	0.507	0.357–0.721	<0.001^*^	1.000	1.386	0.954–2.012	0.086	1.000
Laterality			0.647					
Left	Reference							
Right	1.081	0.773–1.513	0.647	0.983				
Race			0.010^*^				0.277	
White	Reference				Reference			
Black	0.530	0.286–0.983	0.044^*^	1.000	0.538	0.251–1.151	0.110	1.000
Other	0.466	0.228–0.953	0.037^*^	1.000	0.979	0.469–2.046	0.955	0.170
Marital status			0.406					
Married	Reference							
Not married	0.949	0.670–1.346	0.770	0.722				
Unknown	1.627	0.750–3.531	0.218	1.000				
Grade			<0.001^*^				<0.001^*^	
I	Reference				Reference			
II	16512.147	0.000–2.850 ×10^34^	0.785	1.000	11177.452	0.000–3.439 ×10^32^	0.781	1.000
III	166824.855	0.000–2.878 ×10^35^	0.735	1.000	32679.182	0.000–1.006 ×10^33^	0.756	1.000
IV	216497.486	0.000–3.734 ×10^35^	0.729	1.000	46748.769	0.000–1.439 ×10^35^	0.748	1.000
TNM stage			<0.001^*^					
I	Reference							
II	1.964	0.817–4.719	0.131	1.000				
III	11.051	5.495–22.222	<0.001^*^	1.000				
IVA	20.528	10.480–40.212	<0.001^*^	1.000				
IVB	25.339	10.292–62.385	<0.001^*^	1.000				
IVC	127.682	56.104–290.581	<0.001^*^	1.000				
T stage			<0.001^*^				0.002^*^	
T1	Reference				Reference			
T2	2.889	1.557–5.363	<0.001^*^	1.000	1.314	0.685–2.518	0.411	1.000
T3	10.764	6.102–18.988	<0.001^*^	1.000	2.866	1.550–5.300	0.001^*^	1.000
T4a	13.467	7.524–24.105	<0.001^*^	1.000	2.015	1.046–3.881	0.036^*^	1.000
T4b	18.201	7.786–42.548	<0.001^*^	1.000	2.688	1.011–7.144	0.047^*^	1.000
N stage			<0.001^*^				<0.001^*^	
N0	Reference				Reference			
N1	8.221	5.376–12.572	<0.001^*^	1.000	2.513	1.571–4.018	<0.001^*^	1.000
N2a	14.477	4.501–46.559	<0.001^*^	1.000	1.996	0.571–6.983	0.279	1.000
N2b	12.716	8.435–19.171	<0.001^*^	1.000	3.192	2.004–5.084	<0.001^*^	1.000
N2c	15.657	2.156–113.727	0.007^*^	1.000	3.691	0.481–28.309	0.209	1.000
N3	10.499	2.549–43.250	0.001^*^	1.000	2.338	0.521–10.497	0.268	1.000
M stage			<0.001^*^				0.002^*^	
M0	Reference				Reference			
M1	21.597	12.256–38.057	<0.001^*^	1.000	3.554	1.602–7.885	0.002^*^	1.000
Radiotherapy			<0.001^*^				0.763	
No	Reference				Reference			
Yes	3.965	2.669–5.891	<0.001^*^	1.000	1.078	0.661–1.757	0.763	0.998
Chemotherapy			<0.001^*^				0.006^*^	
No	Reference				Reference			
Yes	7.766	5.332–11.311	<0.001^*^	1.000	1.865	1.196–2.906	0.006^*^	1.000
Surgery			<0.001^*^				0.043^*^	
No	Reference				Reference			
Yes	0.080	0.044–0.145	<0.001^*^	1.000	0.423	0.184–0.975	0.043^*^	1.000

**Notes.**

HR hazard ratio CI confidence interval

* indicates *p* < 0.05.

The 5- and 10-year OS consisted of T1 stage (93.3% and 87.3%), T2 stage (86.2% and 75.1%), T3 stage (65.8% and 53.0%), T4 stage (58.7% and 45.4%), N0 stage (90.0% and 81.3%), N1 stage (59.6% and 48.0%), N2 stage (49.2% and 34.6%), and M0 stage (84.3% and 74.6%). Because of the limited number of M1 stage cases, we only observed the 5-year OS (12%). The 5- and 10-year OS also differed greatly between grade I (97.4% and 89.8%), grade II (92.9% and 85.2%), grade III (55.1% and 38.2%), and grade IV (48.8% and 39%).

### Establishment and validation of the nomogram

**Figure 3:**
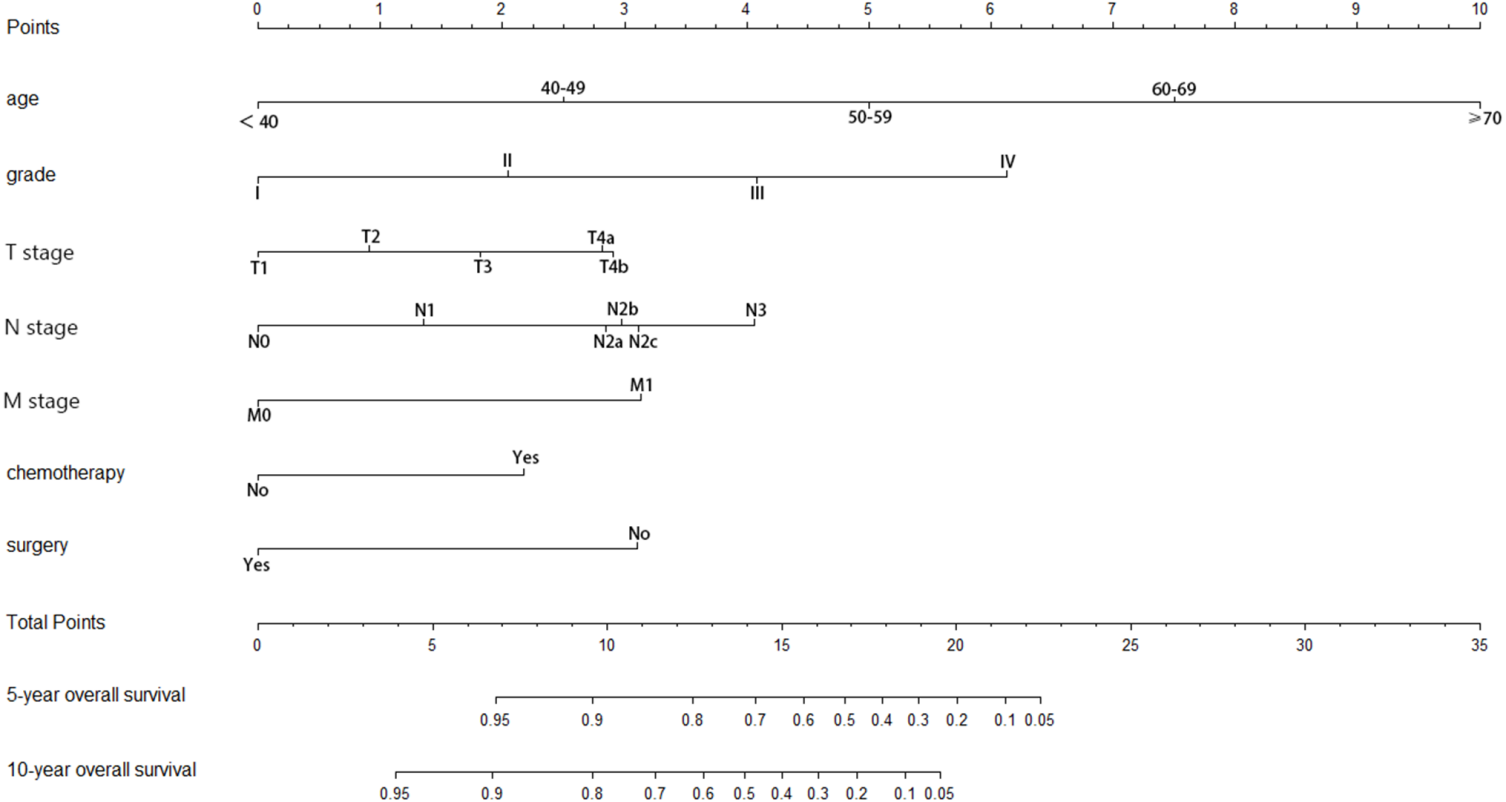
Nomogram of prediction for 5-year and 10-year overall survival.

**Figure 4:**
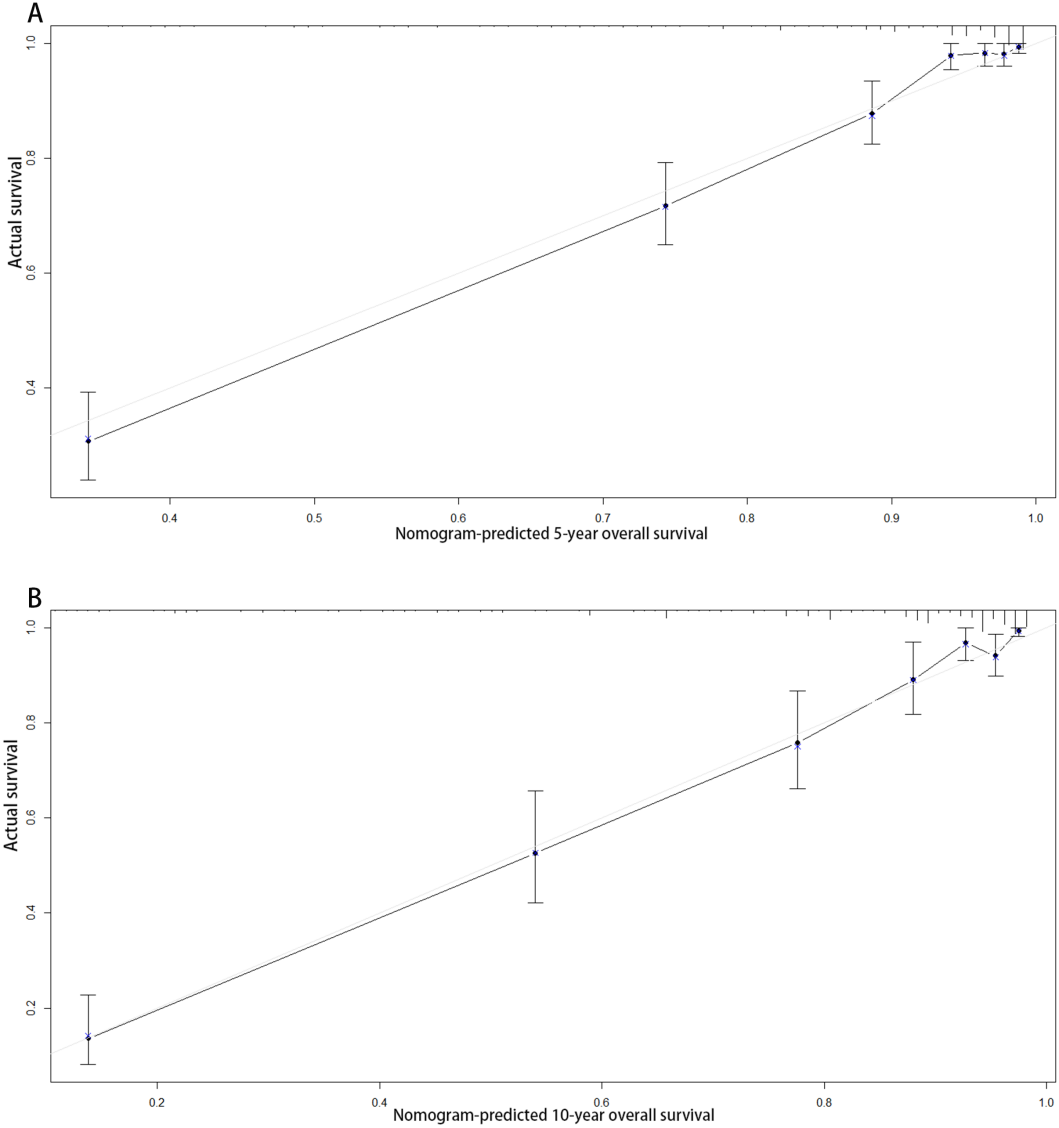
Calibration curves of the nomogram-predicted 5-year (A) and 10-year (B) overall survival.

According to the multivariate analysis, a nomogram for OS was established (Fig. 3). To estimate the 5- and 10-year OS rates, we identified the score for each factor based on the points scale at the top of the nomogram and the sum of the points for each factor. Then, we estimated the 5- and 10-year OS rates based on the points scale at the bottom of the nomogram. The calibration plot based on bootstrap re-sampling validation demonstrated a good agreement between the nomogram-predicted and observed survival rates (Fig. 4). The C-index was 0.8777 (95% CI [0.855–0.898]), suggesting that the nomogram was an accurate model for predicting OS.

## Discussion

MEC accounts for 30%–40% of all malignant tumors of salivary glands. About 70% of MEC in major salivary glands occurs in the parotid gland (Brandwein et al., 2001; Lydiatt et al., 2017). In this study, using more than 1,000 cases from the SEER database, we identified age, grade, T stage, N stage, M stage, chemotherapy, and surgery as independent prognostic factors for both OS and CSS, based on which a nomogram was established to visually and effectively predict the 5- and 10-year OS of patients with P-MEC. To our knowledge, this is the first large-scale population-based study to investigate the prognostic factors and construct a prognostic nomogram that visually and effectively predicts the 5- and 10-year OS of patients with P-MEC. With such a nomogram, we may accurately predict the OS of a P-MEC patient easily with his personalized clinical parameters.

Similar to our results, Bhattacharyya & Fried (2005) reported that the 5-year OS of P-MEC was 81.5%. However, the 10-year OS of P-MEC in their research was 63.5%., which was 73.6% in our study. In our opinion, it may be the reason that they analyzed the survival of patients diagnosed between 1988 and 1998, which was analyzed for patients diagnosed between 2004 and 2015. Undoubtedly, the survival of P-MEC patients were prominently improved with the rapid development of medical science.

Surgical resection remains a cornerstone in the care of patients with P-MEC. In agreement with our results, Rajasekaran et al. reported that the 5-year overall survival was significantly better for patients with surgically resected P-MEC than those with surgery combined with radiotherapy or chemotherapy and without treatment (Rajasekaran et al., 2018). Additionally, they found that patients with increasing age, higher grade, and advanced T, N stage exhibited a worse survival. Bhattacharyya & Fried (2005) also demonstrated that age grade and extraglandular extension. Traditionally, radiotherapy was applied in P-MEC patients with high grade, advanced stage, and unresectable and recurrent tumors. Nevertheless, radiotherapy may result in fatigue and pain, ototoxicity, xerostomia, radiation fibrosis, and radiation-induced malignancy (Chen et al., 2013; Brusic et al., 2012; Zenga et al., 2019). Zenga et al. (2019) reported that P-MEC patients with negative but close (≤2 mm) surgical margins without other high-risk histopathological factors did not benefit from adjuvant radiation in terms of long-term locoregional control. Terhaard et al. (2005) exhibited a significantly improved 10-year local control in T3-4 tumors, close and incomplete resected tumors, and tumors with bone invasion or perineural invasion for patients with malignant salivary gland tumors who received postoperative radiation than those treated with surgery alone. They recommended a dose of at least 60 Gy adjuvant radiation for these patients (Terhaard et al., 2005). In agreement with our research, Bhattacharyya & Fried (2005) also failed to observe the independently prognostic role of radiotherapy for OS in patients with P-MEC. There was little efficacy data for chemotherapy in patients with P-MEC published. It was usually applied as a palliative treatment in patients at advanced stage. We observed that P-MEC patients with chemotherapy exhibited a significantly worse survival. There was still no large series about the efficacy of radiotherapy and chemotherapy for P-MEC, which thus required further researches.

Consistent with our findings, several studies had identified the grade as an important prognostic factor for patients with P-MEC (Rajasekaran et al., 2018; Chen et al., 2014; Bhattacharyya & Fried, 2005). Chen et al. (2014) found that high grade P-MEC was significantly associated with a decreased CSS. Bhattacharyya & Fried (2005) revealed that high grade P-MEC had a significantly worse OS. We also observed that patients with grade III/IV P-MEC had a significantly worse OS than patients with grade I/II tumors.

There were several limitations to this study. First, our study was retrospective with some inevitable bias. Indeed, a larger randomized study is needed to validate our results. Second, the variables which had a great impact on survival to identify the sequence of surgery and chemotherapy and the chemotherapeutic agents with a great impact on survival are not available from the SEER database. Third, other factors may influence the prognosis, such as the surgical margin status, nutritional status, socioeconomic status, drinking and smoking and so on, are not available from the SEER database. Besides, the enrolled patients in our research were all from America, which were mainly the white race. Thus, the reference function of our nomogram may be limited and further comprehensive research is needed to identify these prognostic factors and improve the nomogram.

## Conclusions

We showed that age, grade, T stage, N stage, M stage, chemotherapy and surgery are independent prognostic factors for OS and CSS rates in more than 1,000 American patients with P-MEC. Furthermore, we developed a nomogram that effectively and visually predicts the 5- and 10-year OS in such patients with P-MEC, which could also provide a prognostic reference for other P-MEC patients.

##  Supplemental Information

10.7717/peerj.7237/supp-1 Supplemental Information 1 Survival and prognostic factors of 1306 patients with parotid mucoepidermoid carcinoma

## Additional Information and Declarations

### Data Availability

The following information was supplied regarding data availability: The raw measurements are available as Supplemental File. The raw data shows survival and prognostic factors of 1,306 patients with parotid mucoepidermoid carcinoma.
